# Prediction of the Old-Age Dependency Ratio in Chinese Cities Using DMSP/OLS Nighttime Light Data

**DOI:** 10.3390/ijerph19127179

**Published:** 2022-06-11

**Authors:** Yue Li, Chengmeng Zhang, Yan Tong, Yalu Zhang, Gong Chen

**Affiliations:** 1 Institute of Population Research, Peking University, Beijing 100871, China; liyueleona@foxmail.com (Y.L.); zhangcm@stu.pku.edu.cn (C.Z.); 2101213180@stu.pku.edu.cn (Y.T.); 2 Institute of Ageing Studies, Peking University, Beijing 100871, China

**Keywords:** old-age dependency ratio, nighttime light data, curve regression model, spatial correlation analysis, large and medium-sized cities

## Abstract

The old-age dependency ratio (ODR) is an important indicator reflecting the degree of a regional population’s aging. In the context of aging, this study provides a timely and effective method for predicting the ODR in Chinese cities. Using the provincial ODR from the Seventh National Population Census and Defense Meteorological Satellite Program/Operational Linescan System (DMSP/OLS) nighttime light data, this study aims to predict and analyze the spatial correlation of the municipal ODR in Chinese cities. First, the prediction model of the ODR was established with curve regression. Second, the spatial structure of the municipal ODR was investigated using the Moran’s *I* method. The experimental results show the following: (1) the correlation between the sum of the nighttime light and ODR is greater than the mean of nighttime light in the study areas; (2) the Sigmoid model fits better than other regression models using the provincial ODR in the past ten years; and (3) there exists an obvious spatial agglomeration and dependence on the municipal ODR. The findings indicate that it is reasonable to use nighttime light data to predict the municipal ODR in large and medium-sized cities. Our approach can provide support for future regional censuses and spatial simulations.

## 1. Introduction

China is the country with the largest population of older adults. Along with economic and societal development, population aging becomes complicated and challenging. Since 2000, China has entered an aging society [1]. Its aging population is characterized by a large older population base, aging faster than economic development and significant variations in the level of aging across regions [2].

Population aging refers to the proportion of the old-age population increasing compared to the overall population, accompanied by changes in the age structure. The dependency ratio (DR) is a key indicator to measure the population structure. Therefore, the old-age dependency ratio (ODR) can reflect the degree of aging in a region [3]. The ODR denotes the number of older individuals supported by a working population aged between 15 and 64 years old, which is the fundamental metric used to analyze the population’s age structure and economy [4].

As a result of the aging population and rising ODR, a strain is imposed on the social security system [5]. The rise in the ODR will increase the cost of medical care, cultural entertainment, and living expenses, and reduce the cost of food, clothing, and household devices [6]. Moreover, because the economic, social, and population development exhibit clear regional features, China’s ODR shows a regional imbalance [7]. In this context, predicting the municipal ODR in large and medium-sized cities in China is significant for analyzing the regional structure of the old-age population in China.

There have been relatively few studies predicting and analyzing the municipal ODR in large and medium-sized cities in China. Most scholars indicate the ODR using national population census data from the National Bureau of Statistics [8,9]. The demographic statistics show the distribution characteristics in China’s administrative divisions in terms of population and age structure. Several deficiencies have been identified, such as a high workload and low efficiency. Furthermore, fine-scale census data are scarce. It is detrimental to the research on the fitting of the fine-grained population. Therefore, it is necessary to enhance the traditional ODR prediction approach in large and medium-sized cities in China through more effective spatial analysis.

The ODR reflects the impact of the old-age population on an area’s social and economic development. Understanding the spatial distribution, characteristics, and correlation structure of the ODR has important practical significance for social development and government decision making. Currently, remote sensing images are used as auxiliary data for population estimation and spatial distribution research [10]. In particular, data from remote sensing have the advantages of easy access and timeliness and are not easily affected by natural conditions [11]. With the development of Geographic Information Systems (GIS) and remote sensing technologies, these can be used to analyze and predict the spatial distribution of the population and visualize the degree of population aging. For example, Qiu et al. [12] studied the development models of the urban population using road data and remote sensing images in GIS. It was found that both methods achieve accurate population growth estimates. Using GIS, remote sensing images, and census data, detailed population distribution information in different regions can be obtained at the pixel scale [13]. Therefore, GIS and remote sensing methods have the potential to predict the ODR.

The Operational Linescan System (OLS) sensor carried by the US Defense Meteorological Satellite Program (DMSP) provides a new data acquisition method to research fine-grained population distribution [14]. Compared to data from other conventional remote sensors, DMSP/OLS nighttime light data have the advantages of comprehensive coverage and fast update, allowing it to comprehensively reflect human activities and the regional economy. Currently, DMSP/OLS nighttime light data are commonly utilized to simulate the population density and gross domestic product (GDP) [15,16]. For example, Huang et al. [17] discussed the relationship between DMSP/OLS nighttime light data and the urban population based on three regression models. They established the optimal regression model for simulating the urban population in China. The continuous development of nighttime light research provides a new avenue for population prediction and spatial distribution. Still, they are rarely used to study the municipal ODR in large and medium-sized cities in China. Therefore, there is room to estimate the fine-grained population and examine the spatial distribution using DMSP/OLS nighttime light data.

Using population statistics and DMSP/OLS nighttime light data, this paper predicts the municipal ODR in China, analyzes the spatial distribution in municipal ODR statistics, and determines the pattern of the ODR distribution. The analysis was performed in SPSS statistical software (IBM Corp, New York, NY, USA) using ArcGis 10.5 software tools (Esri Headquarters, Redlands, CA, USA). (1) In light of the few and inaccurate research results on the municipal ODR in large and medium-sized cities in China, the municipal ODR prediction model was presented primarily through the modeling of the ODR and DMSP/OLS nighttime light data; (2) to analyze the spatial correlation of the municipal ODR in Chinese cities, an approach of simulating the spatial distribution of the municipal ODR was proposed based on the spatial autocorrelation analysis; (3) combined with the spatial population data, GDP kilometer grid data, and DMSP/OLS nighttime light data, the spatial distribution characteristics of the municipal ODR in large and medium-sized cities in China were described from the perspectives of the society, economy, and population.

This study makes two significant contributions. First, a new ODR prediction method in large and medium-sized cities in China uses DMSP/OLS nighttime light data to estimate the fine-grained municipal ODR. Second, a spatial autocorrelation analysis method of the municipal ODR in Chinese cities can help analyze the regional aggregation and differences in China’s municipal ODR.

## 2. Background

### 2.1. Nighttime Light Data

The nighttime light image can obtain the silhouette, shape, and structure information of Chinese cities by adjusting the brightness value from low to high. The brightness value of nighttime light is closely related to human and economic activities [18]. Nighttime light imaging is a valuable data source to extract population distribution by investigating the characterization of information on the nighttime light data.

Research mainly includes population estimation and urban population distribution from DMSP/OLS nighttime light data, directly afforded by the remote sensing images [19,20]. For example, Song et al. [21] proposed the dynamic model supported by the Monte Carlo simulation with vegetation-adjusted nighttime light images to map the population of Liaoning province, China. The simulation accuracy was verified using data for 60 counties and 1251 townships. Bagan et al. [22] investigated the spatiotemporal dynamics of expansion by using the gridded land-use data, population census data, and satellite images of nighttime lights. A numerical evaluation of the results showed that the combination of the land-use data and the DMSP/OLS nighttime light data could be used to predict the population density.

Based on the above analysis, nighttime light data are gradually recognized as an essential data source with great potential for predicting population changes. As a key indicator in population research, it is reasonable to estimate the municipal ODR in large and medium-sized cities in China using DMSP/OLS nighttime light data [23]. In addition, most studies find that the spatial differences in the ODR are at the national and provincial scales. However, there are no data available for the municipal ODR studies in some cities. Therefore, through the ODR and nighttime light data, a timely and effective method for predicting the ODR in Chinese cities can be established. The prediction model can provide a new method for census assistance and estimation in some data-poor areas.

### 2.2. Prediction of the ODR

In China, population aging is accelerating. With the size of the expanding old-age population, population aging has become an irreversible trend in the population structure. In the process of population aging, economic and social costs are affected by the rising ODR [24].

Some studies of the ODR depend on the prediction of age- and sex-specific mortality rates, fertility rates, and net migration. The Lee–Carter (LC) method is commonly used in demographic prediction [25]. There are many variants and extensions of the LC method to predict mortality rates and life expectancy [26]. For example, Chowdhury et al. [27] derived a stochastic differential equation age-structured model. One application of the proposed model was in estimating persistence times of age-structured populations, in which variability only results from the birth–death process. Hyndman et al. [28] applied stochastic models to simulate the future age structure of the population to determine the ODR.

Previous studies have focused on predicting the ODR using demographic modeling methods [29]. There are few studies on the prediction of the municipal ODR that further analyze the spatial structure of the municipal ODR. Moreover, most scholars mainly study ODR in a single city case at the municipal scale because of the detailed unpublished data. Therefore, combining the ODR with nighttime light data can more accurately reflect the spatial distribution of the ODR at the municipal scale in China.

### 2.3. Spatial Correlation Analysis of the ODR

In Chinese cities, the spatial distribution of the municipal ODR is highly uneven. This is closely concerned with the development of the economy. The number of old-age individuals varies significantly in different cities. Moreover, the spatial distribution pattern and process of population aging change with time. In the context of an aging population, the spatial distribution of the municipal ODR has specific practical significance.

Spatial autocorrelation is one kind of spatial statistics used to disclose the spatial structure of the regional variable. Some scholars have applied this method to the spatial distribution of the old-age population. For example, Wang et al. [30] proposed an empirical strategy based on spatial autocorrelation methods and spatial error modeling to analyze the spatial patterns of population aging indicators. The results revealed the significant positive spatial autocorrelation and the obvious spatial disparities and the clusters of global aging indicators. Xu et al. [31] studied the spatial distribution characteristics of population aging using the global Moran’s *I* and hotspot analysis and explained the spatial heterogeneity of population aging.

Previous studies have shown the spatial differences in the ODR at the provincial scale [32]. The provinces with a high–high (HH) pattern shift from the eastern to the central provinces. The provinces with a low–low (LL) pattern continuously compress into the northwest provinces. However, there is still research space for the spatial analysis of the fine-grained ODR at the municipal scale. Therefore, comprehensively considering the aspects mentioned above, after predicting the municipal ODR in China using DMSP/OLS nighttime light data, it is of great practical significance to further investigate the spatial structure of the municipal ODR in Chinese cities with the spatial autocorrelation analysis.

## 3. Methods

**Figure 1:**
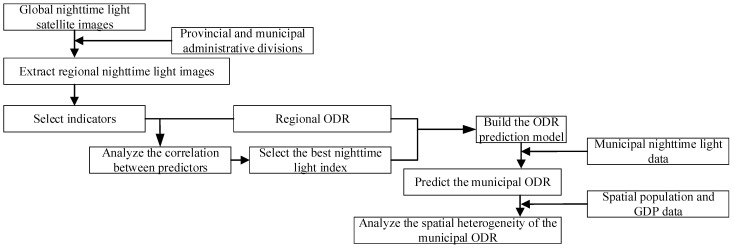
Flowchart of the research method.

To understand the spatial structure of the municipal ODR in Chinese cities, we predicted and analyzed the spatial distribution of the municipal ODR based on DMSP/OLS nighttime light data. This paper selected the provincial administrative regions as the primary administrative units [33]. After excluding the samples with missing data, we obtained 31 provinces and 367 cities. Since the relevant ODR data of Taiwan, Hong Kong, and Macau were not obtained, they were not included in this study. The flowchart is shown in Figure 1.

### 3.1. Data Sources and Preprocessing

#### 3.1.1. Data Sources

The data sources in this study include population age structure and dependency ratios of provinces and cities in China in 2020, DMSP/OLS nighttime light data in China in 2013, spatial population and spatial GDP data in China in 2015, and provincial and municipal administrative divisions in China in 2020. The descriptions of data sources are shown in Table 1. In this study, these data were accessed on 20 January 2022.

The nighttime light data comes from the V4 Version of DMSP/OLS on the National Oceanic and Atmospheric Administration (NOAA) website. The sensor model is F18, which effectively filtered out the background noise of accidental light sources, such as lightning and fishing boats. The width of the light image is 3000 km, and the spatial resolution is 30 arcseconds (1 km × 1 km). The digital number (DN) ranges from 0 to 63. The higher the value, the greater the brightness, meaning the greater the possibility of human activity intensity [34].

Usually, the basic unit of population statistics is the census track area, while the population spatialization replaces the traditional statistics unit with the spatial statistics unit. Based on national population data, the spatial population kilometer grid data comprehensively considers the factors related to population, such as the nighttime light brightness and residential areas [35]. This dataset reflects the detailed spatial distribution of the population in China, and each raster grid data represents the population (people per square kilometer).

GDP is one of the key indicators of social and economic development. The spatialized GDP brings great convenience for data sharing and spatial statistical analysis among regions [36]. The spatial GDP kilometer grid data reflects the detailed spatial distribution of the GDP in China, and each raster grid data represents the total GDP (0.01 million per square kilometer).

#### 3.1.2. Data Preprocessing

**Figure 2:**
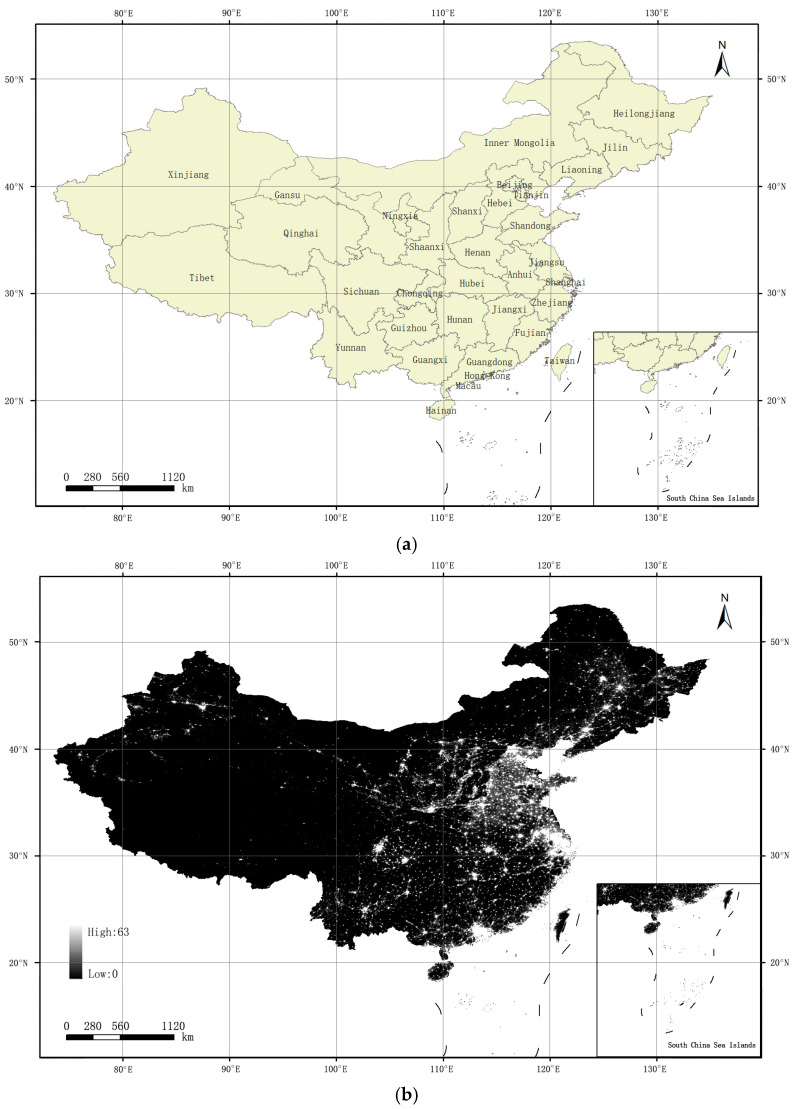
Provincial administrative division map and nighttime light image in China. (**a**) Provincial administrative division map; (**b**) Provincial nighttime light image.

The spatial population and spatial GDP kilometer grid data were extracted by mask. Since the non-radiometric calibration data of DMSP/OLS products are raster images, and the projection format is WGS-84. The original global satellite image was transformed to Albers equal area projection. After the raster image data are projected, the pixel center position usually changes, so it is necessary to resample the input rasters according to certain rules. Resampling is a high-precision method, which can be used to change the spatial resolution of the original remote sensing image. Cubic convolution method is suitable for resampling continuous data. Compared with the rasters obtained by binomial resampling method, cubic resampling method can sharpen the data, and the output rasters have less geometric distortion. So, the cubic technique was used for resampling (the grid size was set to 1 km). Finally, we obtained nighttime light data in China using the mask extraction method. The provincial administrative divisions were extracted, as shown in Figure 2.

### 3.2. ODR Prediction Model Based on the Curve Estimation

#### 3.2.1. Predictors

(1) ODR is an important indicator reflecting the degree of regional population aging from the economic perspective, also known as the old-age dependency coefficient, which refers to the ratio of the old-age population to the working-age population [37], as shown in Equation (1).
  (1) $$ODR=\frac{P_{65^{+}}}{P_{15\sim 64}}\times 100\%$$
  where $P_{65^{+}}$ is the old-age population aged 65 and over; $P_{15\sim 64}$ is the working-age population aged 15~64.

(2) Nighttime light data are closely related to social-economic life and human activity intensity and can reflect the development of population [38]. ODR is a key indicator in population research, and based on the literature review, it is appropriate to use DMSP/OLS nighttime light data to predict the ODR in large and medium-sized cities in China, and thus the study about the relationship information between DMSP/OLS nighttime light data and ODR.

Sum of nighttime light of DN (SUM of DN) and mean of nighttime light of DN (MEAN of DN) can better represent nighttime light changes and the population distribution characteristics in different regions [39,40]. Therefore, the SUM of DN and the MEAN of DN were adopted to estimate the provincial and municipal ODR in this study, as shown in Equations (2) and (3).

(2) $$SUM=\sum_{i=1}^{n}DN_{i}$$

(3) $$MEAN=\frac{SUM}{n}$$

where $DN_{i}$ is the *DN* value of the *i*th effective pixel in the area; *n* is the number of the effective pixels in the area.

#### 3.2.2. Modeling

Due to the differences in regional resources and economic development in China, the provincial ODR values have different distribution characteristics. The quantitative prediction method was used to fit the provincial ODR from the China Statistical Yearbook using the provincial SUM of DN. Then the established model was used to predict the municipal ODR in large and medium-sized cities in China. The curve regression prediction models used in this paper are shown in Table 2. *x* is the input variable; *y* is the output predictor; *b*_0_, *b*_1_, *b*_2_, and *b*_3_ are the constant terms; *μ* is the mean of *x*.

### 3.3. ODR Spatial Correlation Based on the Moran’s I Index

#### 3.3.1. Global Spatial Autocorrelation Theory

Due to the spatial heterogeneity of the municipal ODR in Chinese cities, spatial autocorrelation analysis was used to measure and analyze the degree of dependency among observations in a geographic space, including the global and local spatial autocorrelation [41]. For the global spatial autocorrelation, it is mainly to describe the overall spatial changes and trends among observations. In statistics, Moran’s *I* method was used to measure the spatial autocorrelation. Global Moran’s *I* is defined as:

(4) $$I=\frac{n\sum_{i=1}^{n}\sum_{j=1}^{n}w_{ij}(x_{i}-\bar{x})(x_{j}-\bar{x})}{\sum_{i=1}^{n}\sum_{j=1}^{n}w_{ij}\sum_{i=1}^{n}{\left(x_{i}-\bar{x}\right)}^{2}}$$

where *n* is the number of spatial units indexed by *i* and *j*; *x* is the variable of interest; *x_i_* and *x_j_* are the observations of the *i*th and *j*th units, respectively; $\bar{x}$ is the mean of *x*; *w_ij_* is an element of a matrix of spatial weights.

The spatial weight matrix describes the degree of correlation between provinces and cities and can be divided into the adjacency matrix and distance matrix. In this paper, we selected the adjacency matrix because of the sparseness of points in the region. According to the spatial adjacency relationship, the adjacency Queen matrix was applied because there is no point adjacency in Chinese cities. Therefore, we used Geoda software (an open source software on Github, https://geodacenter.github.io/, accessed on 20 January 2022) for spatial autocorrelation analysis and constructed the weight model based on the Queen contiguity method. The value of Moran’s *I* varies from [−1, 1] (<0: negative correlation; >0: positive correlation; close to 0: there is no spatial autocorrelation). When *I* is close to 1, it indicates that the observations have significant spatial aggregation; otherwise, the spatial distributions of the observations are scattered.

#### 3.3.2. Local Spatial Autocorrelation Theory

Global autocorrelation can describe the overall spatial aggregation of the municipal ODR in Chinese cities. However, it cannot reflect the local spatial correlation and aggregation among observations. Local Indicators of Spatial Association (LISA) was used to judge the local spatial correlation and heterogeneity. The local Moran’s *I* of space unit *i* is defined as Equation (5), and the test statistic of the local Moran’s *I_i_* is Equation (6). *E*(*I_i_*) is the theoretical expectation of Moran’s *I_i_*, *E*(*I_i_*) = $\frac{1}{n-1}$; $\sqrt{VAR\left(I_{i}\right)}$ is the theoretical variance of Moran’s *I_i_*.

(5) $$I_{i}=\frac{n(x_{i}-\bar{x})\sum_{j=1}^{n}w_{ij}(x_{j}-\bar{x})}{\sum_{i=1}^{n}{\left(x_{i}-\bar{x}\right)}^{2}}$$

(6) $$z_{i}=\frac{I_{i}-E(I_{i})}{\sqrt{VAR(I_{i})}}$$

The cold–hot spot analysis is an effective way to explore the local distributions of spatial clusters. Unlike the global Moran’s *I*, Getis-Ord Gi* can reflect the cold–hot spots of observations in the local spatial regions, as shown in Equation (7).

(7) $$G_{i}^{*}=\frac{\sum_{j=1}^{n}w_{ij}x_{j}-\bar{x}\sum_{j=1}^{n}w_{ij}}{S\sqrt{\frac{\left[n\sum_{j=1}^{n}w_{ij}^{2}-{\left(\sum_{j=1}^{n}w_{ij}\right)}^{2}\right]}{n-1}}}$$

In the hypothesis test, we used the *z* value to test the null hypothesis. If −1.96 < *z* < 1.96, *p* > 0.05, it means to accept the null hypothesis; If *z* ≥ 1.96 or *z* ≤ −1.96, *p* ≤ 0.05, it means to reject the null hypothesis, indicating that the spatial correlation between observations is significant; If *z* ≥ 2.58 or *z* ≤ −2.58, *p* ≤ 0.01, it means to reject the null hypothesis, indicating that the spatial correlation between observations is highly significant.

## 4. Results

During the experimental design and simulation process, SPSS software (IBM Corp, New York, NY, USA) was used to predict the provincial and municipal ODR based on the curve regression models, GeoDa software (an open source software on Github, https://geodacenter.github.io/, accessed on 20 January 2022) was used for the spatial autocorrelation analysis of the municipal ODR, and ArcGis 10.5 software (Esri Headquarters, Redlands, CA, USA) was used to analyze the spatial distribution of the municipal ODR in Chinese cities using the ODR from the China Statistical Yearbook, DMSP/OLS nighttime light, spatial population, and spatial GDP grid data.

### 4.1. Analysis of the Prediction Results of the Municipal ODR

According to Appendix A, the prediction model of the ODR was determined. Based on the provincial ODR in China in the past ten years, we compared the prediction results obtained from the curve regression models. Table 3 shows the correlation coefficients (*R*^2^) of different prediction models.

**Figure 3:**
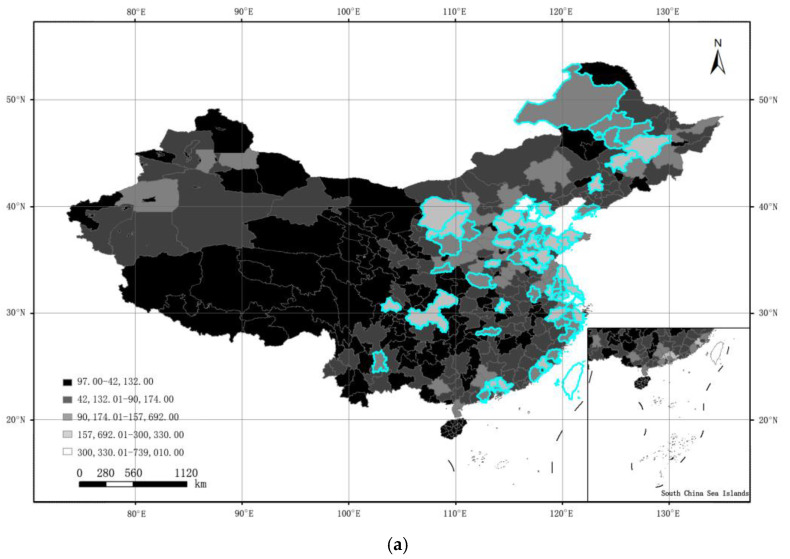

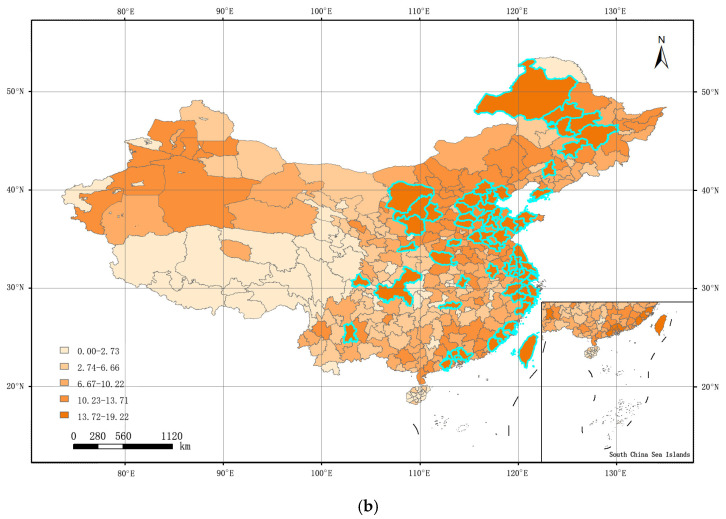
Municipal SUM of DN and municipal ODR in large and medium-sized cities in China. (**a**) Municipal SUM of DN in large and medium-sized cities; (**b**) Municipal ODR in large and medium-sized cities.

From Table 3, although the fitting degrees of all prediction models are not high, the Sigmoid model fits better than other regression models, which confirms that the provincial ODR is related to the provincial SUM of DN. Then, the optimal Sigmoid model was used to predict the municipal ODR in China. Some municipal ODR data are shown in Table 4. To analyze the spatial heterogeneity of the municipal ODR in large and medium-sized cities, from the classification map of the municipal ODR, we set the filter criteria for the municipal ODR ≥ 13.71, and the municipal SUM of DN ≥ 115,762 accordingly. The municipal SUM of DN and municipal ODR in large and medium-sized cities in China are shown in Figure 3. The blue highlighter indicates the selected large and medium-sized cities in China.

As can be seen from Table 4, 65 cities satisfy both the municipal ODR and municipal SUM of DN, and the top three cities are: Suzhou (18.076%), Tianjin (17.923%), and Beijing (17.915%). According to Seventh National Population Census, the old-age population aged over 65 and ODR in these three cities are: Suzhou (1,585,701, 16.806%), Tianjin (2,045,692, 20.555%), and Beijing (2,912,060, 17.678%). Generally, population aging is related to social and economic development [42]. As a result, the size of the old-age population and ODR are growing. Especially since China adopted the policy of reform and opening-up in 1978, life expectancy continued to rise and reached 76.6 years by 2018, notably higher than the world average [43]. The data from Table 4 confirms this point. The top cities are the eastern and central cities with relatively advanced economies, showing that old-age people prefer to receive their benefit pension in large and medium-sized cities.

Recent research has shown that the impact of population migration across regions on the ODR is more apparent. The cities in eastern China have the slowest rate of aging, while those in the midwestern and northeastern regions are growing faster, i.e., the ODR increases rapidly [44,45]. So, some cities with backward economic development have a higher ODR. From Table 4, it is demonstrated that Harbin, the sixth city in the ODR ranking, has a higher ODR (19.557%) than the predicted ODR (17.182%), which is caused by the negative population growth and the outmigration of young people. As the capital of Heilongjiang province, Harbin has a large sum of nighttime light brightness. However, Harbin’s GDP ranks 49th among all cities in China, with only USD 84.1 billion. This conclusion illustrates that the ODR and GDP are contradictory, reflecting a solid link between the ODR and nighttime light brightness.

On the other hand, many studies have shown that nighttime activities are related to economic production activities (especially non-agricultural economic activities) [46]. Moreover, nighttime light data can be a crucial indicator for estimating GDP. The conclusions are presented in Table 4. The cities with a higher ODR also have higher nighttime light brightness. Due to the cohort effect, with further socio-economic development, many immigrants closely related to urban development opportunities will become permanent residents [47]. Hence, cities in eastern China face a severe challenge of population aging. Meanwhile, these cities with higher nighttime light brightness are typically economically developed, and a longer life expectancy will increase the ODR. Although there are differences in the correlations between the ODR, GDP, and nighttime light in several cities, the experimental results show that the ODR is highly correlated with nighttime light data.

### 4.2. Spatial Correlation Analysis of the Municipal ODR

**Figure 4:**
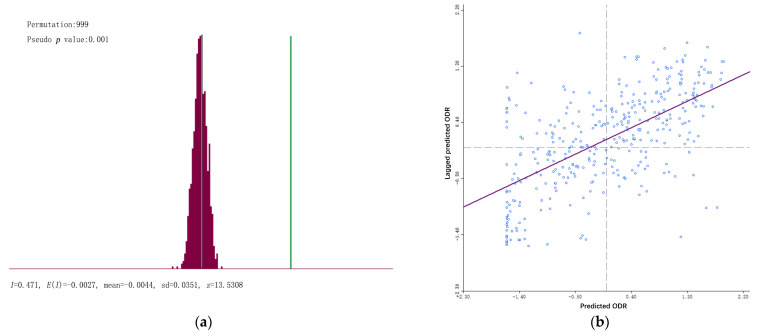
Global spatial autocorrelation analysis of the municipal ODR. (**a**) Significance distribution of the *z* value; (**b**) Scatter plot of the global Moran’s *I*.

Based on the municipal ODR, we analyzed the spatial autocorrelation of the ODR in Chinese cities from the perspective of spatial aggregation. The global spatial autocorrelation metrics of the municipal ODR were calculated through GeoDa software. From Figure 4a, the global Moran’s *I* is 0.471, and the *z* value is 13.5308, indicating that the municipal ODR has a significant autocorrelation in the spatial distribution among cities. Figure 4b shows the scatter plot of the municipal ODR. We can see that these scatters mainly distributed in the first and third quadrants, meaning that the municipal ODR is dominated by the HH and LL clusters. Finally, the global autocorrelation analysis shows a significant spatial correlation and positive impact on the municipal ODR in adjacent cities in China.

**Figure 5:**
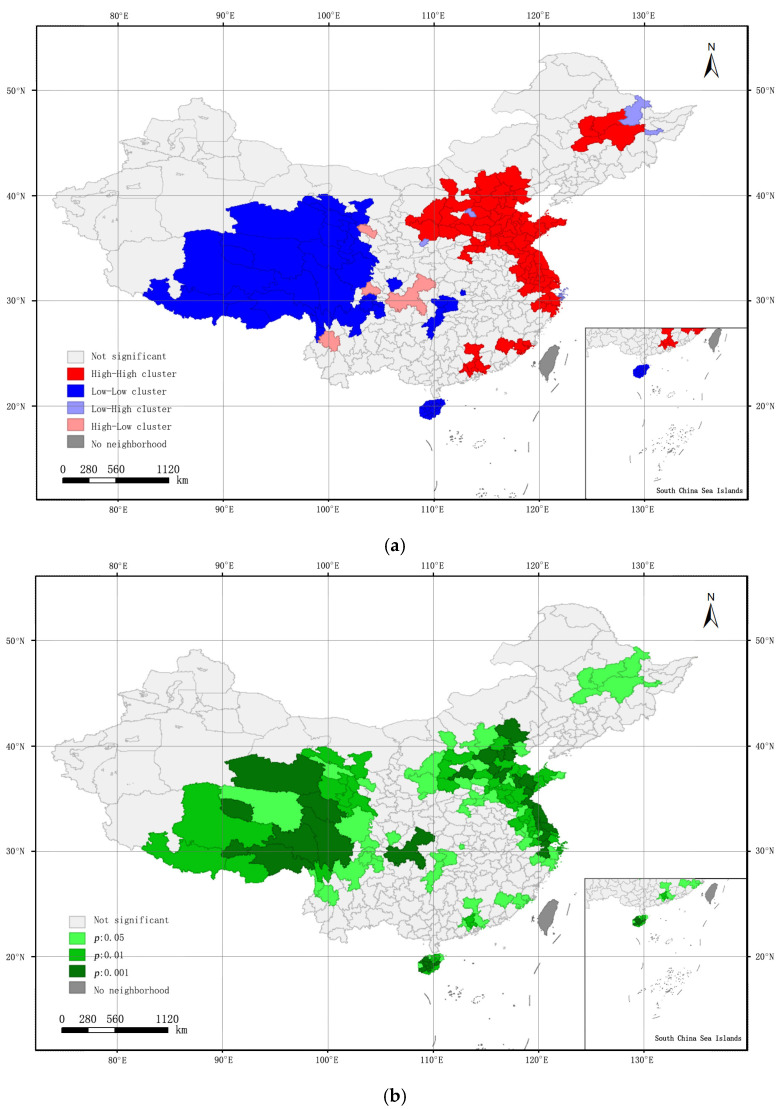

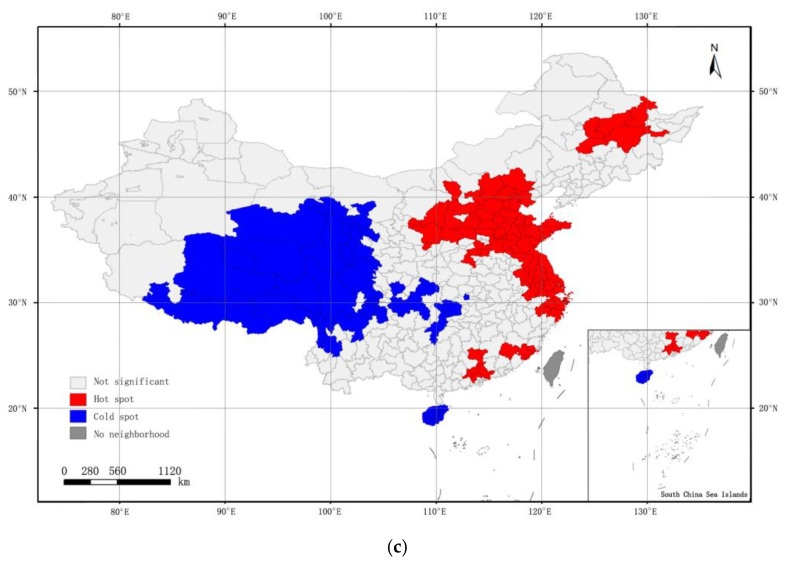
Local spatial autocorrelation and cold–hot spot analysis of the municipal ODR. (**a**) LISA cluster map; (**b**) LISA significance map; (**c**) Gi* cluster map.

In this paper, Global Moran’s *I* was used to analyze the overall positive correlation of the municipal ODR. However, it can’t judge whether there is a positive spatial correlation among the observations. Figure 5 shows the local spatial autocorrelation and cold–hot spot analysis of the municipal ODR, from which we can see the adjacent cities of the HH and LL clusters.

From Figure 5a,b, the local autocorrelation of the municipal ODR is relatively stable. The HH and LL clusters are the main distributions of the municipal ODR in China. The HH distribution is mainly concentrated in the eastern and central regions, while the LL distribution is concentrated in the western region. Based on the significance test of the Moran’s *I* method, it is proved that the aggregation of the municipal ODR is significant. In Figure 5c, we can see the cold–hot spot cities. ODR values in the hot spot cities are shown in Table 5. The statistical results show that there are 84 cities where ODR values are distributed in the hot spot cities. The top cities are Suzhou, Tianjin, Beijing, and Shanghai, which are the large industrial and populous provinces in China.

## 5. Discussion

Nighttime light indices are important spatio-temporal characteristics that reflect human activities, providing a new perspective to reveal the location of the illumination and the extent of human habitation [48,49]. Previous studies have found the application potential of nighttime light in economic and social development [50], but there is little research on nighttime light and the ODR. This study demonstrates the link between nighttime light and the ODR, and the proposed approach can be used to predict the ODR in Chinese cities.

### 5.1. Regional Differences between the ODR and Nighttime Light Data

In the context of China’s aging population, it is necessary to optimize the population and industrial structure to maintain steady economic growth. The population is an important factor affecting economic development, and GDP measures the national economic development level. They play an important role in the future population and overall economic development [51]. As a key indicator to measure population characteristics, the ODR reflects the degree of regional population aging from the perspective of social and economic development. As a result, the relationship between the ODR and nighttime light, population, and GDP is studied. Table 6 shows the correlations between the municipal ODR and the municipal SUM of DN, spatial population, and spatial GDP. The significant correlation coefficients are 0.820, 0.675, and 0.594, respectively.

As shown in Table 6, the ODR is highly correlated with nighttime light and closely related to population and GDP. Previous studies have pointed out that the high ODR values are caused by young people’s outflow rather than being directly affected by economic development [52]. From Table 7, it can be seen that the cities with a high ODR but low GDP are the cities where young people move out. In addition, the contradictions between the ODR, population, and GDP indicate that the current economic situation can be changed. The cities with high ODRs, large populations, and low GDP will face two possibilities. First, the ODR further increases due to young people moving out, which increases the pressure on the old-age care services. Second, young people become the primary local labor force, and population growth drives economic reproduction, thus promoting the rapid growth of GDP.

Based on the above data analysis of the municipal ODR, there are some challenges regarding population aging. Population aging is inevitable for a rapidly expanding economy, and an active response to population aging has been elevated to a national strategy in China [53]. Because of the continuous migration of the population, there are regional differences between “growing rich without growing old” and “growing old before growing rich”. For the regions where young people move in, the cost of immigration is relatively high [54]. However, for the cities where young people move out, it challenges sustainable economic development and puts tremendous pressure on the old-age care systems due to the high ODR. Here, the ODR was used as an indicator reflecting the old-age care services, which cannot reflect the efficiency of the old-age care. In some developed cities with superior medical facilities and efficient care services, the elderly will live in comfort in their old age [55]. In less-developed cities, the rise in the ODR means that the elderly will face a worse quality of life.

Given the cohort effect, the regional imbalance in the municipal ODR makes the old-age care services more challenging in less-developed cities. The fertility boomers of the 1950s and 1960s were primarily born in the underdeveloped towns in the midwest regions. They are facing the crisis of old age and disability [56]. In the past, their children were mainly cared for by elders. Since China introduced the “Family Planning” policy in the late 1970s, the one-child family has become common [57]. The decline in the fertility rate leads to an imbalance in the municipal ODR so that the neo-old-age individuals will face the plight of not receiving care in less-developed cities.

### 5.2. Policy Implications

As the population ages and the ODR increases, the old-age care services face equity and sustainability concerns [58,59]. In particular, the fundamental goal in China is to establish common prosperity. To alleviate the supply–demand imbalance in old-age care services, the 14th Five-Year Plan was proposed to enhance the old-age care services and actively respond to population aging. In 2021, the Central Committee of the Communist Party of China and the State Council issued “Opinions on Strengthening the Work on the Aged in the New Era” to enhance the sense of acquiring happiness and security in one’s old-age. However, more policy suggestions are necessary to improve the well-being of those who are elderly [60]. The findings of this study have three practical policy implications:

(1) Greater emphasis should be placed on the regional variances in the ODR. For example, cities with high nighttime light brightness should develop a local system of old-age care. Attention should also be given to the old-age care in towns with a high ODR where the population has migrated out. Additionally, central financial transfer payments and long-term care insurance should be implemented to ensure the supply of old-age care [61].
(2) To alleviate aging, the elderly should be immersed in the activities of the common prosperity objective where they can discover their self-worth through time banking programs [62]. As the capital with a relatively high ODR, Beijing officially launched the “Project of Time Banking for Elderly Services” in 2022. Studies have shown that elderly individuals can build their social capital by participating in community life, allowing them to independently and happily age in peace.
(3) Family support policies could also alleviate the regional disparity in the ODR. China implemented the “Three-Child Policy” in 2021. Due to the cohort effect, this policy would not be conducive to solving the current pension crisis and will raise the social dependency ratio [63,64]. As a result, it is critical to implement suitable strategies to address the rapidly changing demographics. For example, for young talents, policymakers could consider providing them with more favorable policies that allow them to cohabit with their parents. The income disparity between regions can be decreased through industrial transfer and other measures for young workers.

## 6. Conclusions

The aging of the population has become a severe social problem. Therefore, it is crucial to investigate the relationship between an aging population and economic growth. Predicting the ODR in metropolitan cities could assist China in addressing the issues associated with an aging society. On the other hand, counting the municipal ODR in large and medium-sized cities in China requires time and effort.

This study establishes the municipal ODR prediction and spatial correlation analysis using DMSP/OLS nighttime light data. The distribution characteristics and spatial differences of the municipal ODR were analyzed in Chinese cities, which can provide suggestions and references for estimating the regional ODR and restraining aging population. The main conclusions can be summarized are as follows:

First, for the municipal ODR prediction model, by comparing the curve regression models, it is determined that the fitting effect between the sum of the nighttime light data and the ODR is better than the mean of nighttime light data. Using the provincial ODR in the past ten years, this study found that the Sigmoid model is suitable for predicting the municipal ODR. It also confirms that the municipal ODR is highly correlated with the sum of the nighttime light data. Second, for the spatial autocorrelation analysis based on the Moran’s *I* method, the results show a significant spatial dependence on the municipal ODR in Chinese cities. The local autocorrelation is relatively stable.

## 7. Further Research

However, the research on the prediction methods of the ODR using nighttime light data is still a bottleneck. There may be an ODR bias using only nighttime light, and the prediction accuracy of the ODR needs to be further improved. In future research, we will further optimize and compare the prediction models to recommend policy interventions to mitigate the influence of aging on the economic development of municipalities in China.

## Figures and Tables

**Table 1:** Descriptions of data sources.

Name	Source	Information
Population age structure and dependency ratios	National Bureau of Statistics of China (http://www.stats.gov.cn/tjsj/ndsj/2021/indexch.htm) accessed on 20 January 2022	xls format
Global DMSP/OLS nighttime light data	NOAA official data center (https://ngdc.noaa.gov/eog/dmsp/downloadV4composites.html) accessed on 20 January 2022	TIF format, WGS-84 projection
Spatial population and spatial GDP grid data	Resource and environment science and data center (https://www.resdc.cn/) accessed on 20 January 2022	Gird format, Albers projection
Provincial and municipal administrative divisions	National geomatics center of China (http://www.ngcc.cn/) accessed on 20 January 2022	Shapefile format

**Table 2:** Curve regression models.

No.	Model	Expression	No.	Model	Expression
1	Linear	$y=b_{0}+b_{1}x$	7	Power	$y=b_{0}(x^{b_{1}})$
2	Logarithmic	$y=b_{0}+b_{1}\ln(x)$	8	Sigmoid	$y=e^{b_{0}+b_{1}/x}$
3	Inverse	$y=b_{0}+\frac{b_{1}}{x}$	9	Growth	$y=e^{b_{0}+b_{1}x}$
4	Quadratic	$y=b_{0}+b_{1}x+b_{2}x^{2}$	10	Exponential	$y=b_{0}e^{b_{1}x}$
5	Cubic	$y=b_{0}+b_{1}x+b_{2}x^{2}+b_{3}x^{3}$	11	Logistic	$y=\frac{1}{1/\mu +b_{0}b_{1}{}^{x}}$
6	Compound	$y=b_{0}b_{1}{}^{x}$			

**Table 3:** Correlation coefficients (*R*^2^) of different prediction models using the provincial ODR in China in the past ten years.

Model	ODR_2011	ODR_2012	ODR_2013	ODR_2014	ODR_2015	ODR_2016	ODR_2017	ODR_2018	ODR_2019	ODR_2020	Mean
Linear	0.043	0.031	0.029	0.050	0.064	0.067	0.076	0.135	0.121	0.075	0.069
Logarithmic	0.120	0.089	0.084	0.119	0.164	0.179	0.165	0.213	0.192	0.206	0.153
Inverse	0.177	0.153	0.171	0.169	0.238	0.280	0.226	0.234	0.217	0.302	0.217
Quadratic	0.062	0.038	0.030	0.061	0.100	0.106	0.102	0.144	0.130	0.141	0.091
Cubic	0.259	0.190	0.205	0.230	0.225	0.218	0.191	0.252	0.217	0.257	0.224
Compound	0.058	0.043	0.038	0.073	0.068	0.076	0.077	0.129	0.115	0.073	0.075
Power	0.170	0.127	0.123	0.178	0.201	0.234	0.198	0.253	0.228	0.244	0.196
Sigmoid	0.254	0.221	0.254	0.254	0.314	0.398	0.301	0.326	0.304	0.412	0.304
Growth	0.058	0.043	0.038	0.073	0.068	0.076	0.077	0.129	0.115	0.073	0.075
Exponential	0.058	0.043	0.038	0.073	0.068	0.076	0.077	0.129	0.115	0.073	0.075
Logistic	0.058	0.043	0.038	0.073	0.068	0.076	0.077	0.129	0.115	0.073	0.075

**Table 4:** Prediction results of the municipal ODR in large and medium-sized cities in China.

No.	City	Municipal SUM of DN	Municipal ODR (%)	No.	City	Municipal SUM of DN	Municipal ODR (%)	No.	City	Municipal SUM of DN	Municipal ODR (%)
1	Suzhou	373,261	18.076	23	Wuxi	186,016	15.948	45	Jiaxing	145,137	14.865
2	Tianjin	349,408	17.923	24	Yulin	180,977	15.838	46	Xi’an	144,576	14.847
3	Beijing	348,231	17.915	25	Nanjing	180,621	15.830	47	Dongguan	140,280	14.702
4	Shanghai	334,746	17.819	26	Zhengzhou	176,195	15.728	48	Yan’an	140,273	14.701
5	Chongqing	300,330	17.538	27	Shijiazhuang	174,421	15.686	49	Jinhua	139,724	14.682
6	Harbin	265,159	17.182	28	Jinan	173,470	15.663	50	Taizhou	139,068	14.659
7	Guangzhou	256,275	17.078	29	Shenyang	170,688	15.595	51	Qiqihar	138,330	14.633
8	Tangshan	254,191	17.053	30	Handan	170,627	15.593	52	Xingtai	136,448	14.566
9	Weifang	249,205	16.990	31	Jining	170,199	15.582	53	Yangzhou	136,258	14.559
10	Ningbo	232,518	16.765	32	Wuhan	170,083	15.580	54	Binzhou	136,179	14.556
11	Nantong	230,225	16.731	33	Foshan	169,524	15.566	55	Taizhou	135,832	14.543
12	Chengdu	223,101	16.624	34	Huizhou	167,721	15.520	56	Hulunbeir	135,027	14.513
13	Yantai	218,269	16.548	35	Xuzhou	166,657	15.492	57	Luliang	133,380	14.452
14	Qingdao	214,348	16.483	36	Dalian	157,692	15.249	58	Changzhou	133,260	14.447
15	Linyi	211,939	16.443	37	Fuzhou	156,327	15.210	59	Jiangmen	132,327	14.412
16	Quanzhou	210,609	16.420	38	Kunming	155,533	15.187	60	Hefei	132,237	14.409
17	Hangzhou	208,367	16.381	39	Nanyang	152,235	15.089	61	Heze	131,710	14.388
18	Cangzhou	200,063	16.230	40	Langfang	150,066	15.022	62	Dezhou	130,769	14.352
19	Changchun	197,844	16.188	41	Daqing	149,761	15.013	63	Shaoxing	126,333	14.174
20	Baoding	189,983	16.032	42	Suihua	146,411	14.907	64	Changsha	123,222	14.043
21	Yancheng	188,884	16.009	43	Wenzhou	145,549	14.879	65	Dongying	115,762	13.706
22	Ordos	187,564	15.981	44	Zhangzhou	145,227	14.868				

**Table 5:** ODR values in the hot spot cities.

No.	City	Municipal ODR (%)	No.	City	Municipal ODR (%)	No.	City	Municipal ODR (%)	No.	City	Municipal ODR (%)
1	Suzhou	18.076	22	Shijiazhuang	15.686	43	Heze	14.388	64	Zhongshan	12.231
2	Tianjin	17.923	23	Jinan	15.663	44	Dezhou	14.352	65	Hengshui	12.216
3	Beijing	17.915	24	Handan	15.593	45	Shaoxing	14.174	66	Pingdingshan	12.103
4	Shanghai	17.819	25	Jining	15.582	46	Dongying	13.706	67	Datong	11.513
5	Harbin	17.182	26	Foshan	15.566	47	Xinxiang	13.596	68	Chengde	11.051
6	Guangzhou	17.078	27	Huizhou	15.520	48	Lianyungang	13.574	69	Zaozhuang	10.961
7	Tangshan	17.053	28	Xuzhou	15.492	49	Xinzhou	13.430	70	Xiamen	10.823
8	Weifang	16.990	29	Langfang	15.022	50	Zhenjiang	13.404	71	Rizhao	10.822
9	Ningbo	16.765	30	Daqing	15.013	51	Liaocheng	13.404	72	Longyan	10.550
10	Nantong	16.731	31	Suihua	14.907	52	Huai’an	13.372	73	Kaifeng	9.968
11	Yantai	16.548	32	Jiaxing	14.865	53	Zibo	13.281	74	Putian	9.827
12	Qingdao	16.483	33	Dongguan	14.702	54	Weihai	13.164	75	Songyuan	9.614
13	Linyi	16.443	34	Yan’an	14.701	55	Suqian	13.162	76	Shaoguan	9.588
14	Quanzhou	16.420	35	Jinhua	14.682	56	Linfen	13.122	77	Ma’anshan	9.550
15	Cangzhou	16.230	36	Taizhou	14.659	57	Hohhot	12.910	78	Puyang	9.351
16	Baoding	16.032	37	Xingtai	14.566	58	Jinzhong	12.822	79	Xuancheng	8.701
17	Yancheng	16.009	38	Yangzhou	14.559	59	Chuzhou	12.660	80	Yichun	6.896
18	Wuxi	15.948	39	Binzhou	14.556	60	Huzhou	12.623	81	Zhoushan	6.095
19	Yulin	15.838	40	Taizhou	14.543	61	Changzhi	12.496	82	Qitaihe	5.565
20	Nanjing	15.830	41	Luliang	14.452	62	Zhangjiakou	12.471	83	Yangquan	4.734
21	Zhengzhou	15.728	42	Changzhou	14.447	63	Tai’an	12.466	84	Tongchuan	2.040

**Table 6:** Correlations between the municipal ODR and nighttime light SUM of DN, spatial population, and spatial GDP.

	Municipal ODR	Municipal SUM of DN	Spatial Population	Spatial GDP
Municipal ODR	1			
Municipal SUM of DN	0.820 **	1		
Spatial population	0.675 **	0.674 **	1	
Spatial GDP	0.594 **	0.702 **	0.816 **	1

Note: ** significant correlation at 0.01 level (two-sided).

**Table 7:** Distribution of the municipal ODR and municipal SUM of DN, spatial population, and spatial GDP.

City	Municipal ODR (%)	City	Municipal SUM of DN	City	Spatial Population	City	Spatial GDP
Suzhou	18.076	Suzhou	373,261	Chongqing	30,959,389	Shanghai	261,830,605
Tianjin	17.923	Tianjin	349,408	Shanghai	23,876,188	Beijing	217,296,830
Beijing	17.915	Beijing	348,231	Beijing	21,690,535	Tianjin	187,427,119
Shanghai	17.819	Shanghai	334,746	Tianjin	15,425,497	Guangzhou	180,167,046
Chongqing	17.538	Chongqing	300,330	Chengdu	15,220,175	Chongqing	147,182,175
Harbin	17.182	Harbin	265,159	Guangzhou	13,439,208	Suzhou	133,678,096
Guangzhou	17.078	Guangzhou	256,275	Yichun	12,562,974	Chengdu	96,746,196
Tangshan	17.053	Tangshan	254,191	Baoding	11,946,975	Qingdao	91,469,755
Weifang	16.990	Weifang	249,205	Suzhou	10,738,576	Changsha	90,364,523
Ningbo	16.765	Ningbo	232,518	Wuhan	10,568,583	Wuxi	86,232,644
Nantong	16.731	Nantong	230,225	Linyi	10,410,237	Hangzhou	86,143,887
Chengdu	16.624	Chengdu	223,101	Handan	10,220,940	Yangzhou	85,564,657
Yantai	16.548	Yantai	218,269	Nanyang	10,100,919	Wuhan	80,912,736
Qingdao	16.483	Qingdao	214,348	Shijiazhuang	10,077,521	Foshan	80,506,132
Linyi	16.443	Linyi	211,939	Harbin	9,278,484	Dongguan	79,439,884
Quanzhou	16.420	Quanzhou	210,609	Weifang	9,275,444	Nanjing	76,500,052
Hangzhou	16.381	Hangzhou	208,367	Wenzhou	9,063,728	Ningbo	76,441,765
Cangzhou	16.230	Cangzhou	200,063	Qingdao	8,989,079	Shenyang	70,454,732
Changchun	16.188	Changchun	197,844	Zhoukou	8,903,463	Dalian	69,840,050
Baoding	16.032	Baoding	189,983	Heze	8,793,076	Zhengzhou	69,838,666

**Figure A1:**
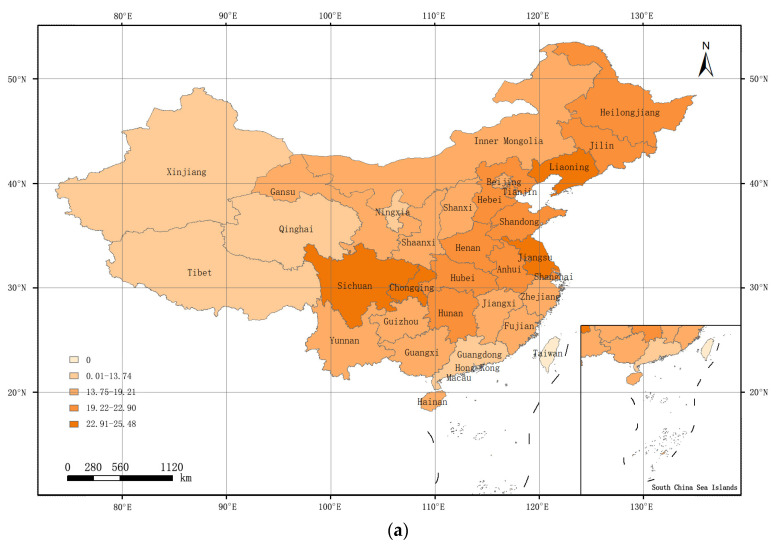

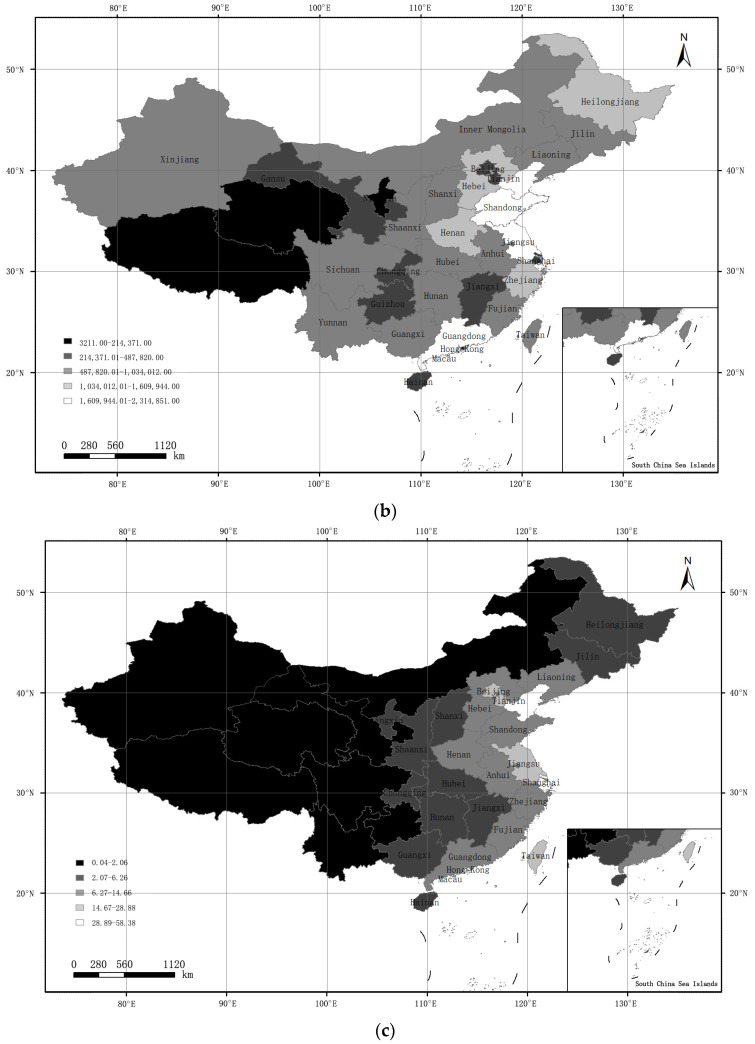
Real provincial ODR, provincial SUM of DN, and provincial MEAN of DN. (**a**) Real provincial ODR; (**b**) Provincial SUM of DN; (**c**) Provincial MEAN of DN.

**Figure A2:**
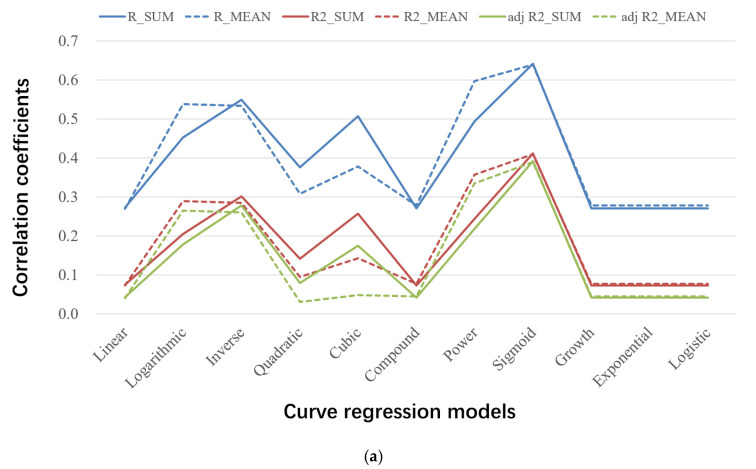

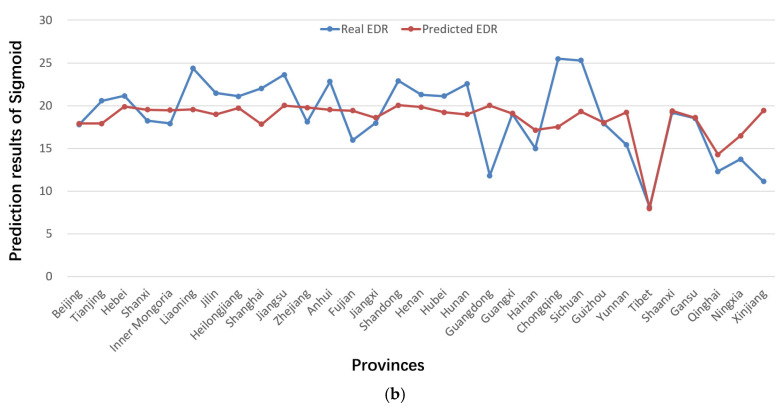
Fitting effects of different models. (**a**) Correlation coefficients; (**b**) Prediction results of Sigmoid.

## Data Availability

Data employed in this study are public data with sources provided in the article.

## Appendix A. Modeling Process

According to the mask extraction of the provincial administrative divisions in China, the natural discontinuity classification method (Jenks) was used to divide the predictor values into five levels, and we reduced the decimal places in legends. Figure A1 shows the real provincial ODR, provincial SUM of DN, provincial and MEAN of DN. Based on Equations (2) and (3), we calculated the nighttime light variables to reflect the provincial ODR in China. Meanwhile, ODR values from the China Statistical Yearbook were counted to compare the correlation between the provincial SUM of DN and provincial MEAN of DN, and the results are shown in Table A1.

**Table A1:** Pearson correlations between ODR and nighttime light variables.

Province	Provincial SUM of DN	Provincial MEAN of DN	Provincial ODR (%)	Province	Provincial SUM of DN	Provincial MEAN of DN	Provincial ODR (%)
Beijing	348,231	21.22	17.77	Hunan	614,523	2.90	22.56
Tianjin	349,408	28.88	20.56	Guangdong	2,076,629	11.43	11.82
Hebei	1,609,944	8.54	21.14	Guangxi	661,632	2.79	19.01
Shanxi	981,743	6.26	18.24	Hainan	260,939	5.99	14.99
Inner Mongolia	941,327	0.82	17.90	Chongqing	300,330	3.65	25.48
Liaoning	1,034,012	6.98	24.37	Sichuan	804,188	1.65	25.28
Jilin	612,406	3.21	21.47	Guizhou	362,748	2.06	17.92
Heilongjiang	1,263,697	2.79	21.08	Yunnan	750,280	1.96	15.42
Shanghai	334,746	41.95	22.02	Tibet	49,183	0.04	8.13
Jiangsu	2,171,169	20.95	23.61	Shaanxi	862,672	4.20	19.21
Zhejiang	1,361,539	12.69	18.10	Gansu	487,820	1.15	18.50
Anhui	980,574	7.00	22.83	Qinghai	129,387	0.19	12.31
Fujian	881,809	7.06	15.95	Ningxia	214,371	4.13	13.74
Jiangxi	483,089	2.89	17.97	Xinjiang	874,442	0.54	11.12
Shandong	2,314,851	14.66	22.90	Mean value	850,516.26	7.80	18.83
Henan	1,500,920	9.06	21.28	Correlation coefficient	0.260 *	0.258 *	
Hubei	747,395	4.02	21.11	Significance of the correlation coefficients	0.040	0.041	

Note: * significant correlation at 0.05 level (two-sided).

The Kendall correlation coefficient was used to assess the consistency of data. The analysis results show that the correlation coefficient between the provincial SUM of DN and ODR is higher than that between the provincial MEAN of DN and ODR. Although the correlation coefficients are not very high, the significances of the correlation coefficients were verified, indicating that the prediction results are statistically significant. Therefore, the provincial SUM of DN was used as a fitting index to construct the prediction model of the municipal ODR in large and medium-sized cities in China.

In SPSS software, we selected the provincial SUM of DN as the independent variable and the provincial ODR as the dependent variable. The fitting results of the eleven regression models are shown in Table A2. The provincial MEAN of DN was set as the independent variable in the comparative experiment, and the fitting results are shown in Table A3.

**Table A2:** Summary and parameter estimates for all models (the provincial SUM of DN as an argument).

		Summary							Parameter Estimates			
No.	Model	*R*	*R* ^2^	Adjusted *R*^2^	*F*	df1	df2	Sig.	Constant	b_1_	b_2_	b_3_
1	Linear	0.273	0.075	0.043	2.336	1	29	0.137	17.143	1.985 × 10^−6^		
2	Logarithmic	0.453	0.206	0.178	7.501	1	29	0.010	−11.977	2.304		
3	Inverse	0.549	0.302	0.278	12.542	1	29	0.001	20.421	−6.493 × 10^5^		
4	Quadratic	0.376	0.141	0.080	2.303	2	28	0.119	14.716	8.328 × 10^−6^	−2.788 × 10^−12^	
5	Cubic	0.507	0.257	0.175	3.117	3	27	0.043	10.412	2.958 × 10^−5^	−2.682 × 10^−11^	7.021 × 10^−18^
6	Compound	0.271	0.073	0.041	2.296	1	29	0.141	16.488	1.000		
7	Power	0.494	0.244	0.218	9.381	1	29	0.005	2.336	0.154		
8	Sigmoid	0.642	0.412	0.392	20.336	1	29	0.000	3.019	−4.644 × 10^4^		
9	Growth	0.271	0.073	0.041	2.296	1	29	0.141	2.803	1.205 × 10^−7^		
10	Exponential	0.271	0.073	0.041	2.296	1	29	0.141	16.488	1.205 × 10^−7^		
11	Logistic	0.271	0.073	0.041	2.296	1	29	0.141	0.061	1.000

**Table A3:** Summary and parameter estimates for all models (the provincial MEAN of DN as an argument).

		Summary							Parameter Estimates			
No.	Model	*R*	*R* ^2^	Adjusted *R*^2^	*F*	df1	df2	Sig.	Constant	b_1_	b_2_	b_3_
1	Linear	0.270	0.073	0.041	2.280	1	29	0.142	17.857	0.125		
2	Logarithmic	0.538	0.289	0.265	11.812	1	29	0.002	16.657	1.609		
3	Inverse	0.534	0.285	0.260	11.553	1	29	0.002	19.490	−0.511		
4	Quadratic	0.309	0.095	0.031	1.473	2	28	0.246	17.155	0.316	−0.005	
5	Cubic	0.378	0.143	0.048	1.505	3	27	0.236	15.893	0.890	−0.049	0.001
6	Compound	0.278	0.077	0.045	2.430	1	29	0.130	17.179	1.008		
7	Power	0.597	0.357	0.335	16.095	1	29	0.000	15.757	0.109		
8	Sigmoid	0.640	0.410	0.389	20.126	1	29	0.000	2.953	−0.038		
9	Growth	0.278	0.077	0.045	2.430	1	29	0.130	2.844	0.008		
10	Exponential	0.278	0.077	0.045	2.430	1	29	0.130	17.179	0.008		
11	Logistic	0.278	0.077	0.045	2.430	1	29	0.130	0.058	0.992		

Two sets of data were analyzed with the curve regression methods, from Table A2 and Table A3, we can see that the Logarithmic, Inverse, Power, and Sigmoid models are credible (Sig. < 0.05). Moreover, the correlation between the provincial SUM of DN and provincial ODR is better than the provincial MEAN of DN for all models. Figure A2 shows the correlation coefficients of all models and the provincial ODR fitting curve of Sigmoid. The results of various regression analyses can be made out, and the Sigmoid model fits better than other regression models (*R* = 0.642, *R*^2^ = 0.412, adjusted *R*^2^ = 0.392, Sig. = 0.000). Therefore, the prediction model of the ODR was set: $y=e^{3.019-46439.179/x}$.
